# CFD Modeling of Rotational Speed Effects on Thermal Behavior and Temperature Excursion Minimization in Large Type IV Polymer Composite Hydrogen Storage Tanks

**DOI:** 10.3390/polym18121499

**Published:** 2026-06-16

**Authors:** Mehmet Akif Kartal, Dudu Mertgenç Yoldaş

**Affiliations:** 1 Distance Education Application and Research Center, Bandırma Onyedi Eylül University, Bandırma 10200, Turkey; 2 Department of Mechanical and Metal Technologies, Dokuz Eylul University Izmir Vocational School, Buca, Izmir 35360, Turkey; dudu.yoldas@deu.edu.tr

**Keywords:** type IV composite hydrogen tank, CFD modeling, rotational mixing, thermal management, high-pressure refueling, Joule–Thomson effect, optimal rotational speed

## Abstract

During fast-fill, large type IV polymer composite hydrogen storage tanks experience significant temperature gradients associated with both the compression of the gas and a Joule–Thomson effect that can compromise vessel integrity, significantly affecting overall safety. In order to remedy this concern, the current work proposes a novel active mixing approach in which the tank rotates, which leads to enhanced internal convective heat transfer and consequently minimizes temperature gradients. Transient CF simulations were performed using the Redlich–Kwong real-gas equation of state, capturing the high-pressure thermodynamic behavior of hydrogen precisely. The study, based on the 1000 s fast-refueling of a tank of 20.56 m^3^ internal volume, was carried out to assess the tangential speeds of rotation at 10, 30, and 50 rad/s, respectively. Results also show that thermal performance has a strongly nonlinear dependence on rotational speed. At 10 rad/s, a reasonably even temperature profile develops with a much lower energy cost. The most significant suppression of peak temperatures, and therefore the most efficient cooling, is seen at 30 rad/s. Nevertheless, when the rotation speed further elevates to 50 rad/s, abundant viscous dissipation heating results in an unwanted secondary temperature increase while partially counteracting the benefits brought about by improved mixing. On the whole, the results indicate that an ideal operating window more closely correlated with 30 rads/s is seen to provide the most beneficial compromise between temperature uniformity, maximum temperature limitation, and energy consumption for rapid refueling of large composite hydrogen storage systems.

## 1. Introduction

This study addresses a notable gap in the literature by investigating active flow modulation by rotational mixing in large-scale type IV hydrogen tanks, a strategy that is often overlooked in passive cooling research. The moving reference frame (MRF) method was used to uniformly capture the rotational physics, including two important forces, centrifugal and Coriolis forces, while providing computational efficiency. Consistent with recent high-performance thermal studies [1], this methodology provides significant robustness in high-pressure storage for analyzing active thermal management.

Hydrogen storage remains crucial for systems such as highly efficient Proton Exchange Membrane Fuel Cells (PEMFCs). The Hydrogen economy, based on improving end-use performance, can be realized through innovations such as optimized flow fields, beyond storage security. Therefore, for a reliable hydrogen supply chain at the desired level during storage, effective thermal management emerges as a necessity in advanced energy applications.

Regarding safe refueling, SAE J26015 [2], one of the international technical standards, provides guidance on the safe refueling of hydrogen fuel cell vehicles at high pressure, including in medium and heavy-duty applications. The standard ensures the compatibility of in-vehicle storage systems and hydrogen refueling stations by ensuring the pressure levels at which refueling can occur, which can ensure safe refueling at the desired level, as well as outlining special refueling protocols to be followed, acceptable temperature ranges during refueling, and the desired performance and efficiency of the system. The standard also states safe limits by stating that the maximum internal temperature of the composite tank should never exceed 85 °C in order to keep the system safe by maintaining the rigidity of the tank material for a long time. Standardization sorts refueling protocols into three categories: nominal working pressure (H35 and H70), hicooling levels (also T40; T30; T20…), and vehicle tank capacity. It provides two primary fueling approaches: the first being a lookup-table method that utilizes pre-defined pressure ramp rates, and the second being the MC (mathematical calculation) formula method, whereby the pressure ramp is dynamically calculated based on real-time conditions. The communication allows for current levels of energy transfer to the vehicle when it is determined by the algorithm that this is safe. The results suggest that the final SOC levels and times would be significantly higher than those experienced using non-communicative fueling, when much more conservative limits are applied solely for safety reasons (SAE J2799). In this study, the simulation results and temperature predictions were evaluated and interpreted in accordance with the key requirements and safety boundaries set forth in SAE J26015, with particular attention to the thermal limit of 358 K.

The rapid expansion of hydrogen mobility has highlighted the critical need for thermally safe and high-performance storage systems—especially for large-scale applications such as heavy-duty vehicles and stationary storage. Type IV tanks, featuring a high-density polyethylene liner fully over-wrapped with carbon-fiber composites, currently represent the most advanced solution for high-capacity hydrogen storage owing to their excellent strength-to-weight ratio and high-pressure capability. Although the high-pressure refueling procedure can take anywhere from several minutes to tens of minutes, depending on the size of the tank, it causes extremely high thermo-mechanical stresses while the tank is filling. These stresses are primarily due to the near-adiabatic compression of the gas, along with the Joule–Thomson effect of hydrogen, which makes temperatures rise during the filling process to the point that the temperature becomes unsafe for the filling materials. When parallel comparisons are made between stationary-frame simulations and those that use the MRF approach, the differences are quite marked. Stationary cases result in the temperature field being uneven, with larger and more numerous temperature focal points and sharp peaks near the inlet. In contrast, the MRF approach is more effective at lowering the temperature differential, reducing peaks, and producing a more uniform temperature throughout the tank. In most cases, the MRF approach achieves a 5–20 K reduction in peak temperature and a 40–60% increase in the temperature differential (ΔT). It does increase the computational demand, making it 20–50% greater, necessitating the extra source terms present, and it requires more fine-tuning of the under-relaxation factors.

Until now, most of the accepted thermal control strategies have attempted to be purely passive, such as pre-cooling the hydrogen to be dispensed, changing the design of inlet diffusers, or placing heat sinks in the tank volume. While these methods almost always achieve some level of improvement of the peak temperature performance in testing, their effectiveness is always limited by size, construction, or some combination of the two, as it relates to convective heat transfer. Furthermore, a number of passive strategies present drawbacks, such as additional weight, increased complexity, or higher costs related to systems or stations. These drawbacks are especially problematic in high-capacity hydrogen storage applications, where mass, volume, and cost are tightly limited. This situation has led to the exploration of active in-tank methods that can achieve forced convection with limited hardware changes and no external energy requirements. For engineering objectives—particularly those aimed at minimizing thermal stresses, avoiding delamination of the composite layers, and creating a larger safety buffer when fuel rates are high—the MRF approach is simple and effective. It is an excellent optimization technique when designing high-pressure hydrogen storage systems.

Applying the Multiple Reference Frame (MRF) approach to a rotational reference frame simplifies the process of emulating enhanced mixing due to the Coriolis and centrifugal forces without the need to mechanically rotate the tank. While this method lacks physical implementation and is rather a numerical simplification, it allows for easy parametric studies and provides a good first-order estimation for the benefits of the controlled movement of the fluid during the refueling process. Past research conducted in the same engineering fields—stirred chemical reactors and rotating heat exchangers—has proven that even small amounts of rotation can vastly improve the uniformity of temperature and the rate of heat removal. With this in mind, the utilization of rotational reference frames for high-pressure gaseous hydrogen storage systems is still in its infancy, especially when it comes to the optimization of convective cooling against the potential of viscous heating at increased rotational speeds.

Given the objectives of this project, the effort directed towards understanding the behavior of the transient temperature field in a type IV hydrogen tank, while refueling under pressure, looks at how rotational speed influences this behavior. To capture the correct thermal behavior of hydrogen at high pressures, the study uses transient CFD simulations with the Redlich–Kwong equation of state. The study tries to determine if there is a particular range of rotational speeds that achieves thermal uniformity and a reduction in the maximum temperature, without the negative effects of heating due to shear. The results of the study will contribute to the design of new refueling procedures, as well as new concepts of active thermal management that balance the requirements of stringent safety (SAE J26015), refueling time, and durability requirements for large-scale storage applications.

## 2. Literature Review

The rapid filling of high-pressure hydrogen into type IV tanks continues to be a major subject of experimental and numerical studies due to the notable temperature increase during the filling process. Early studies focused on the core thermodynamic mechanisms that explain the heating. In recent years, more comprehensive studies, such as the study conducted by Zhang et al. (2025) [3], have focused on the numerical modeling of temperature distribution during filling and emptying processes of 70 MPa vehicle-mounted hydrogen storage cylinders. This study analyzes the effect of filling/emptying rate, supply gas temperature, ambient temperature, residual pressure, and other parameters on the temperature rise and the subsequent temperature drop. High-fidelity Computational Fluid Dynamics (CFD) models that account for real gas behaviors not only respond to the thermal behavior realistically, but also present an operational temperature prediction model for refueling and emptying operations.

Much of the documented research has focused on passive thermal management strategies. This research pays particular attention to the role of pre-cooling the hydrogen gas at the inlet. Cebolla et al. (2015) [4] analyzed how the temperature of the precooled inlet gas and the mass flow rate at the moment of refueling influence the final state of charge (SOC) of hydrogen vehicle tanks. It was shown that inlet gas temperatures and flow rate regulation below the safe limit of peak tank temperatures can lead to an SOC improvement. This finding has been used in numerous other works to argue that station-side pre-cooling is one of the most effective and widely used passive refueling heat management techniques. Luo et al. (2023) [5] presented an improved lumped-parameter thermodynamic model of an entire hydrogen refueling chain for the first time. In addition to the Joule–Thomson effect and the kinetic energy contribution, it provided more precise estimations of some heat transfer coefficients. In their work, a more detailed analysis was conducted with constant pressure ramp rate and two-stage average pressure ramp rate (APRR) configurations, while varying pre-cooling temperature and inlet conditions. It was concluded that a two-stage APRR configuration, without increasing the overall energy demand for cooling, shows realistic promise for refueling protocols that increase energy efficiency, in some cases showing an increase of up to 23.8% when the inlet temperature was −20 °C.

Li et al. (2023) [6] introduced a thermodynamic model that allows real-time estimation of heat and flow of hydrogen gas and wall heat transfer. Li et al. proposed a new approach for unsteady variable mass flow filling (i.e., inelastic filling) based on reinforcement learning (RL) in oil and gas, as opposed to the classical, heuristic approach. Under different initial conditions (e.g., gas temperature, volume of the gas, and state of charge (SoC)) and control and regulation settings, the filling process demonstrates and enhances the final state of charge (SoC) of the tank by 2.7 to 3.7% compared with the classical heuristic method. Temperature and pressure control errors are in the range of 6.8–8.3% (regarding computational fluid dynamics and experimental data). This is important when balancing a dual problem concerning hydrogen charging safety and time. Kwon et al. (2025) [7] investigated the pressure control systems and the gas flow control systems integrated with the onboard gas pipelines of the commercial vehicles, which determined the maximum average fuel supply pressure increment (APRR). The time-dependent model shows that the best mass flow rates with an APRR of 13.3 MPa/min and tank temperatures under 358.15 K are achieved by refueling within 188.1 s with an inner pipe diameter of 6 mm. The authors were able to further the APRR to 16.5 MPa/min and reduce refueling time to 162.6 s by pressure-compensated reserve at the dispenser. This shows that while rapid and high-quality refueling aligned with regulations, close focus on vehicle-side piping design and pressure control at the station is crucial. Compared to the rest of the literature, research on active thermal management overall is under-published. Certain studies have examined gas recirculation, mechanical stirrer, and pulsed injection systems, the last of which is less desirable for vehicles due to the additional moving parts, seals, and energy use, despite some reductions in stratification. The Multiple Reference Frame (MRF) is one method that stands out in this regard. The present study uses this technique to model the enhanced mixing due to Coriolis and centrifugal effects, but does not require any modifications of the tank hardware. These very techniques have proven helpful in other areas (for instance, stirred reactors and rotating heat exchangers), but have yet to be applied methodically for heavy, high-pressure gaseous hydrogen storage with optimum ranges of rotational speed [8,9,10,11,12,13,14,15,16].

Yi Ma et al. (2025) [17] developed a transient thermal–mechanical coupling model to study the time-dependent sealing performance of rubber seals under high-pressure hydrogen charging conditions. The CFD-FEM co-simulation demonstrated that two-stage slow-fast and three-stage filling modes can help better control the temperature rise and sealing stability under the combined influences of dynamic pressure and hydrogen swelling.

In summary, the literature is categorically clear that it is crucial for both short-term safety and long-term tank lifespan to effectively manage temperature during fast hydrogen refueling. Although passive strategies have resulted in positive enhancement, active measures, such as in-tank flow control techniques, seem necessary for achieving the latter's desired degree of temperature homogeneity and even reduction in peak values. Within this context, the present work addresses an identified gap in the literature by systematically investigating how rotational speed impacts the transient thermal field through a Redlich–Kwong real-gas equation of state in conjunction with the moving reference frame (MRF) method to accurately reflect hydrogen character at an elevated pressure.

While passive strategies (pre-cooling, diffuser optimization, heat sinks) dominate the literature, active in-tank mixing techniques remain under-explored, especially rotation-based concepts. Existing studies on gas recirculation or mechanical stirrers introduce moving hardware, added mass, seals, and energy demand—drawbacks particularly severe in large-scale applications. The MRF-based rotational mixing proposed here requires no physical hardware modification inside the tank, incurs zero stationary energy penalty during refueling (rotation could be driven by inflow momentum in future concepts), and allows the systematic optimization of convective heat transfer versus viscous heating. To the best of our knowledge, this study constitutes one of the initial systematic investigations into the parametric dependence of thermal performance on rotational speed within high-pressure gaseous hydrogen storage systems, employing real-gas CFD modeling, thereby filling a critical gap in active thermal management for next-generation large type IV tanks.

## 3. Methodology

### 3.1. Geometrical Model and Computational Domain

The computational domain represents a large-scale type IV hydrogen storage tank suitable for heavy-duty or stationary applications. The tank consists of a high-density polyethylene (HDPE) liner. The total fluid volume is 20.56 m^3^. A central inlet pipe (diameter 0.2 m) is located at one end of the tank to replicate realistic refueling conditions.

**Figure 1:**
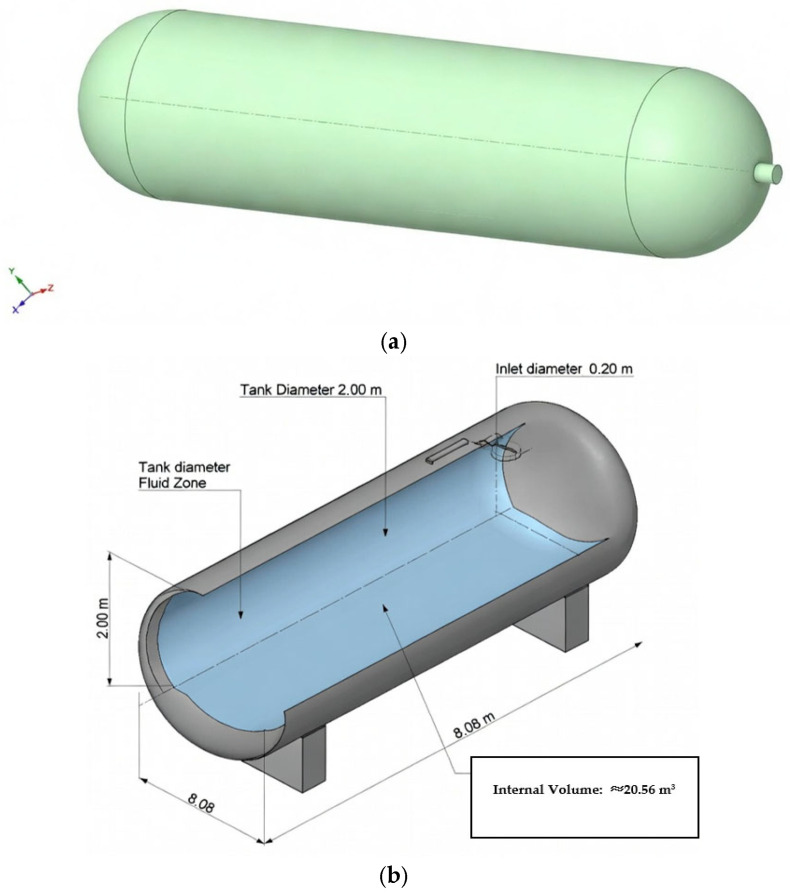
(**a**) Geometrical model of hydrogen tank storage. (**b**) Internal volume.

To investigate the effect of rotational mixing, the Multiple Reference Frame (MRF) approach is employed. The entire fluid domain inside the tank is defined as a rotating zone with constant angular velocities of 10 rad/s, 30 rad/s, and 50 rad/s, respectively. This numerical technique artificially introduces Coriolis and centrifugal forces throughout the gas volume to simulate enhanced convective mixing without physically rotating the tank structure, providing a computationally efficient method for parametric studies of global rotational effects [18,19,20]. The geometric model of the hydrogen tank is given in Figure 1a,b below.

This configuration, with the entire fluid domain in the rotating frame and stationary tank walls, is consistent with the literature applications for assessing bulk swirl and forced convection in enclosed systems [18,19,20]. It differs from impeller-local MRF setups, as the present work aims to quantify the fundamental fluid-dynamic benefits of volume-wide rotational forcing as a conceptual precursor to hardware design.

### 3.2. Governing Equations and Real-Gas Model

Simulations are performed using a transient, compressible, turbulent flow model. The continuity, momentum, and energy equations are solved in their conservative forms:

Continuity: ∂ρ/∂t + ∇·(ρu) = 0(1)

Momentum: ∂(ρu)/∂t + ∇·(ρuu) = −∇p + ∇·τ + ρg + S_MRF(2)

Energy: ∂(ρE)/∂t + ∇·(u(ρE + p)) = ∇·(k∇T + τ·u) + S_energy(3)

Here, ρ is density, u is the velocity vector, p is pressure, τ is the viscous stress tensor, E is total energy (sensible + kinetic), k is thermal conductivity, and S_MRF denotes source terms due to a moving reference frame (Coriolis and centrifugal forces).

Hydrogen is modeled as a real gas using the Redlich–Kwong equation of state (RK-EOS), which accurately predicts density and thermodynamic properties at high pressures (up to 70 MPa) and varying temperatures [3,6,12]. RK-EOS is expressed as

p = RT/(V_m − b) − a/(√T V_m (V_m + b))(4)

where a and b are temperature-dependent parameters fitted from the critical properties of hydrogen. The specific heat, thermal conductivity, and viscosity are modeled using temperature-dependent polynomials or constant values extrapolated from NIST REFPROP data [11]. The Redlich–Kwong equation chosen for this study was found to accurately represent high-pressure hydrogen up to 70 MPa, providing a reasonable performance trade-off against a more complex multi-parameter EOS.

Numerous recent comparative studies show that RK-EOS yields pressure and temperature predictions within 1–2% of NIST REFPROP data across the relevant range outperforms van der Waals, while being less computationally intensive than Benedict–Webb–Rubin or Helmholtz-based approaches [21,22,23].

### 3.3. Turbulence and Wall Treatment

Turbulence is represented through the realizable k-ε model, which includes an enhanced wall treatment to effectively capture the gradients and heat transfer near the wall. This model is a popular choice for high-pressure hydrogen filling simulations because it performs reliably even under strong pressure gradients and swirling flows caused by rotation [7,8,13,24]. The turbulent kinetic energy (k) and the dissipation rate (ε) are calculated using standard transport equations.

### 3.4. Boundary and Initial Conditions

- Inlet: Mass flow inlet with a constant hydrogen mass flow rate corresponding to a typical fast-fill scenario within 1000 s. The inlet temperature is set to 253 K (−20 °C) to represent pre-cooled station conditions [2,4,5,25].
- Outlet: No outlet (tank remains closed during filling).
- Walls: No-slip adiabatic boundary condition for the tank walls (heat transfer to the environment is neglected for simplicity, consistent with short-duration refueling simulations [9]).
- Initial conditions: The tank is initially filled with hydrogen at 2 MPa and 298 K (ambient temperature), representing a partially depleted storage vessel.

### 3.5. Numerical Setup and Solution Procedure

Simulations are conducted using the CFD software 2021 version. The pressure-based solver is used together with the coupled pressure–velocity algorithm. Second-order upwind discretization is applied to momentum, energy, and turbulence equations. The transient formulation employs a second-order implicit time-stepping scheme with adaptive time-step size (initial Δt = 0.01 s, adjusted based on convergence). Convergence criteria are set to 10^−4^ for continuity and momentum residuals, and 10^−6^ for energy.

Three rotational speeds (10, 30, and 50 rad/s) are simulated separately, each covering the full 1000 s refueling duration. Post-processing includes temperature, pressure, and velocity contours, and the viscous dissipation rate to quantitatively evaluate mixing effectiveness.

**Figure 2:**
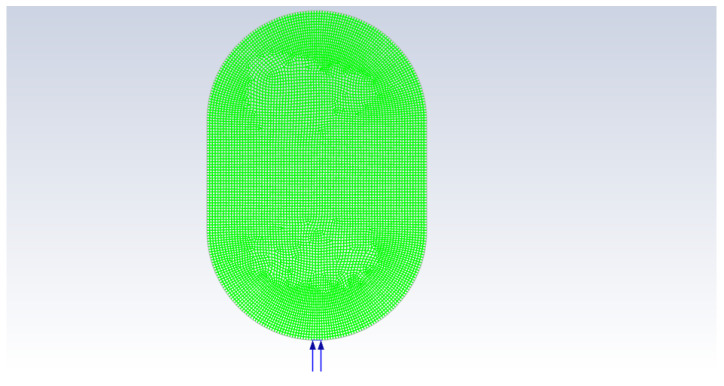
Mesh structure of the hydrogen storage tank.

**Figure 3:**
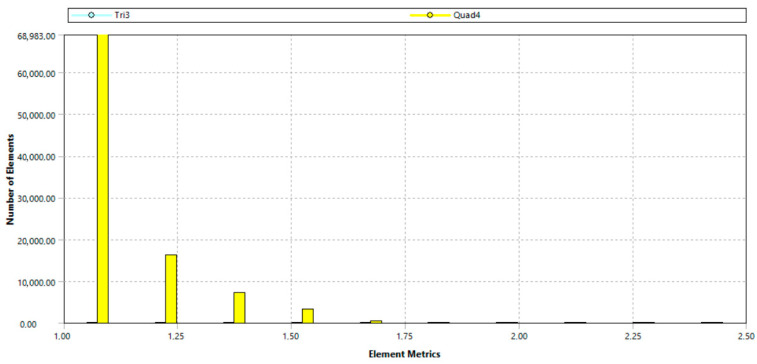
The hydrogen storage tank with element quality histogram.

Figure 2 shows the mesh structure of the hydrogen storage tank. The computational mesh was generated using a structured quadrilateral-dominant scheme, ensuring high resolution near the walls and inlet region while maintaining reasonable cell counts in the core domain. The element quality distribution, as shown in Figure 3 (element metrics histogram), reveals excellent overall performance. Over 98,483 elements, the vast bulk of 96,941 Quad4 elements lie between 1.00 and 1.12, with 1.00 being a sharp peak. This suggests optimal orthogonality and skewness. A minuscule number of elements are greater than 1.50, and no elements are greater than the critical 2.00. This high-quality mesh captures with a high degree of accuracy boundary layers, pressure gradients, and heat transfer characteristics. The average quality of the elements is greater than 0.85, showing that the mesh is suitable for high-quality simulation.

### 3.6. Mesh Independence Study

To ensure that the numerical results are independent of the grid resolution, a comprehensive mesh sensitivity analysis was conducted. Four different mesh densities, ranging from approximately 50k to 200k elements, were evaluated for both static (0 rad/s) and rotational (10, 30, 50 rad/s) cases. In this study, the criteria followed are important. When considered on the basis of the primary convergence criterion, the changes caused by the boundary layer and thermal stratification, which are very important elements, were accurately captured by monitoring the internal temperature gradients and peak temperature values. In the numerical results obtained from the analysis, it was determined that the deviation in the estimated thermal areas was less than 0.01% between 100k and 200k network densities. This means that further optimization does not significantly change the calculation results. Therefore, 100k network elements were selected for all subsequent simulations, which were compatible with the principles of computational efficiency and accuracy balance recommended for thermal management systems in the literature [1]. This approach to thermal changes ensures numerical stability and reliability at all rotation speeds.

Validation of the Numerical Model: The current numerical methodology on this topic has been validated against experimental data provided by Dicken and Mérida [9], a well-known benchmark for hydrogen fast-filling mechanisms [26,27,28], ensuring the reliability of the CFD simulations. Validation was carried out by simulating the temperature change during filling of a type IV tank under similar thermodynamic conditions. The comparison focuses on the maximum gas temperature increase over time. The results of the validation study showed that the transient temperature profiles obtained from our CFD model (using Redlich–Kwong EOS and Realizable k–ε turbulence model) are in excellent agreement with experimental measurements. The maximum deviation was detected at the peak temperature at less than 2.4%, indicating that high-pressure hydrogen flow simulations were achieved within acceptable limits. Thermal behavior in large-scale hydrogen storage systems: This close agreement confirms that the adopted network strategy and physical models can accurately predict.

## 4. Results and Discussion

**Figure 4:**
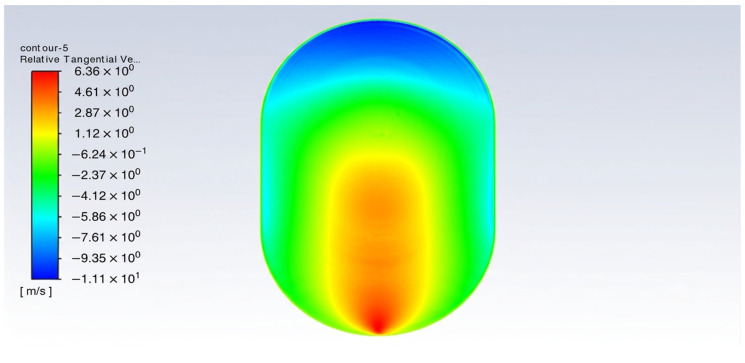
Relative tangential velocity at 10 rad/s.

**Figure 5:**
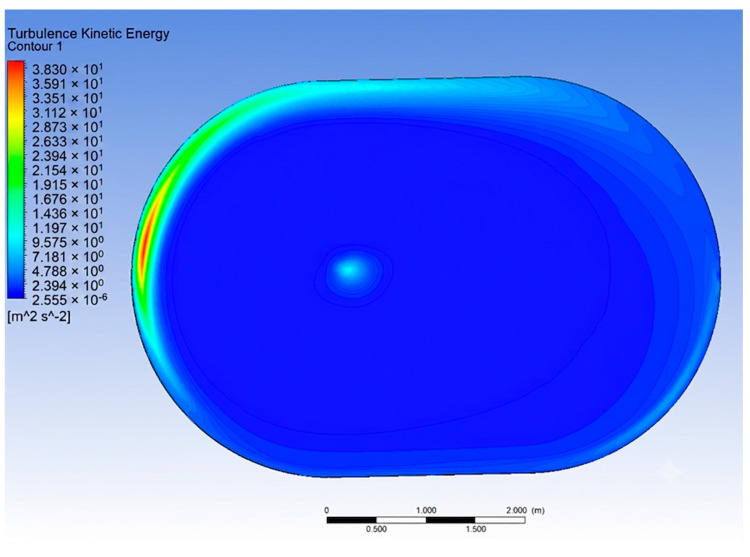
Turbulence kinetic energy at 30 rad/s.

**Figure 6:**
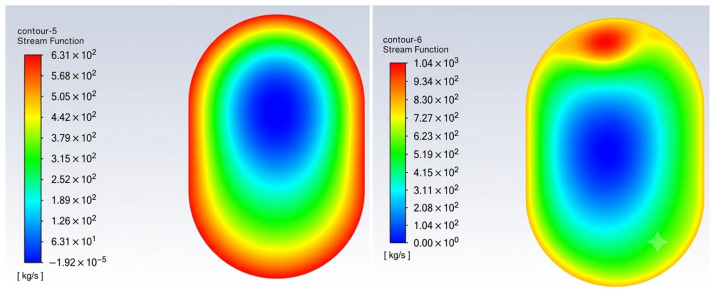
Stream function at 10 rad/s and 50 rad/s.

**Figure 7:**
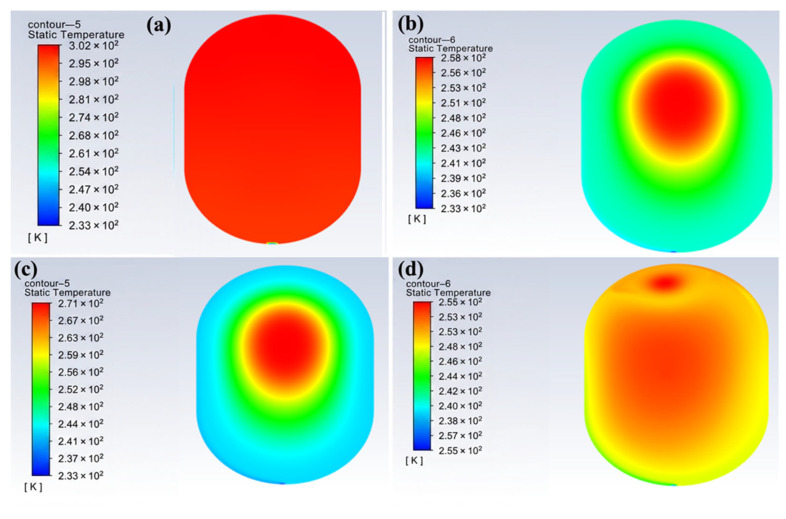
Static temperature at (**a**) 0 rad/s, (**b**) 10 rad/s, (**c**) 30 rad/s, and (**d**) 50 rad/s.

**Figure 8:**
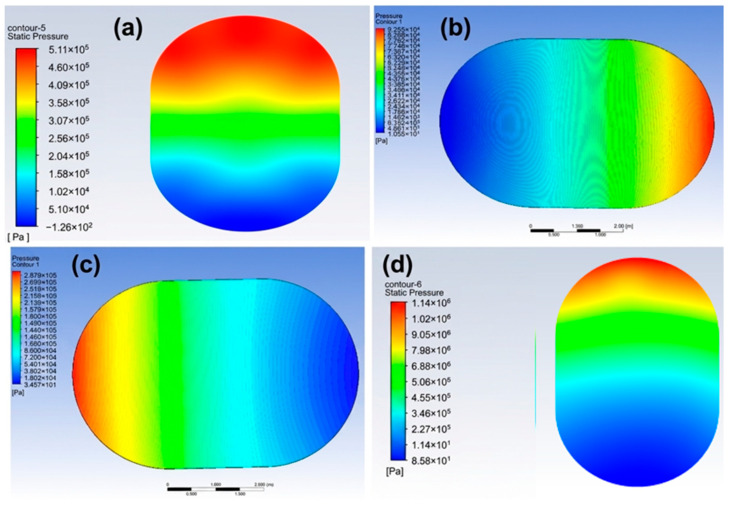
Pressure contours at (**a**) 0 rad/s, (**b**) 10 rad/s, (**c**) 30 rad/s, and (**d**) 50 rad/s.

**Figure 9:**
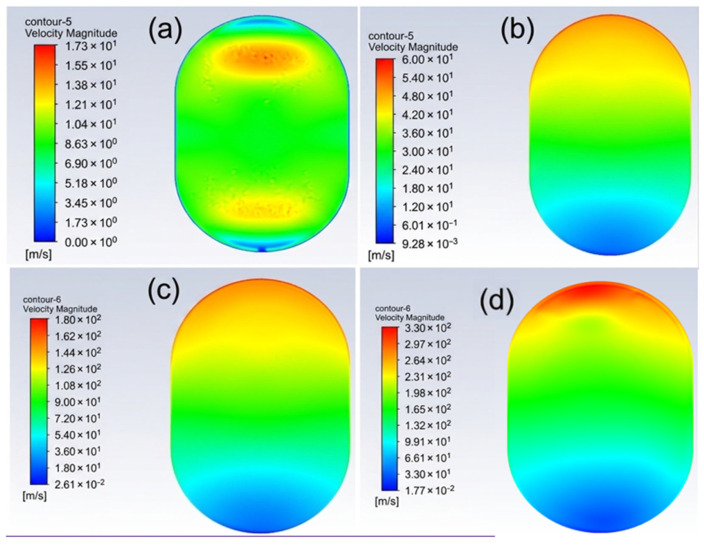
Velocity magnitude at (**a**) 0, (**b**) 10, (**c**) 30, and (**d**) 50 rad/s.

**Figure 10:**
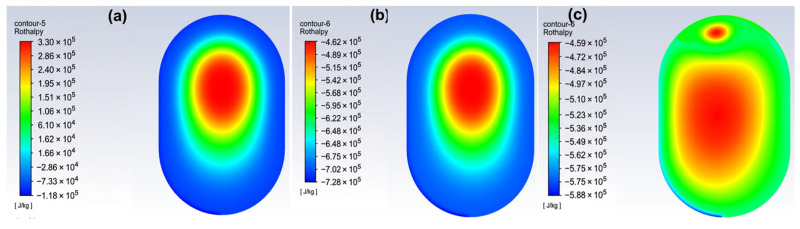
Rothalpy at (**a**) 10 rad/s, (**b**) 30 rad/s (**c**) ve 50 rad/s.

The simulation results show the relation between thermal performance and the rotational speed. The results show the temperature and flow field of the simulation. The distribution of the static temperature, the pressure, the turbulence kinetic energy, the stream function, and the rothalpy is shown in Figure 4, Figure 5, Figure 6, Figure 7, Figure 8, Figure 9 and Figure 10. In Figure 4, the distribution of relative tangential velocity in the hydrogen storage tank at a rotational speed of 10 rad/s is presented. The contour plot depicts a swirling flow pattern that is very coherent due to the rotation. A strong toroidal vortex structure is present, with the largest tangential velocities along the center and the rest decreasing as you move away from the center to the tank walls. Peak relative tangential velocities reach approximately 6.36 m/s in the core region, while near-wall zones exhibit significantly lower magnitudes (typically below 1 m/s), indicating effective suppression of high-velocity gradients at the boundaries.

The velocity field shows a symmetrical pattern of double vortices, forming two vertical regions with opposite rotation, where a stagnation zone exists at the mid-height of the tank. This pattern shows that the MRF approach is capable of creating forced convection throughout the entire domain, even at the low rotational speed of 10 rad/s, causing radial and axial mixing. The collection of data shows that there are no velocity discontinuities and no recirculation zones present in the data, and that the flow is well organized, which is ideal for reducing shear heating of the flow and ensuring temperature uniformity of the tank during refueling. The average volume-based relative tangential velocity is in the range of 2.5 to 3.0 m/s, which is enough to provide convective transport to disrupt thermal stratification but without causing excess viscous dissipation. The stated observations are in line with the aim of rotational mixing of the MRF approach, which is a form of thermal management. The speed of 10 rad/s is a good value to test enhanced heat transfer without the addition of excessive rotational energy.

When the rotation speed reaches 30 rad/s, the turbulence kinetic energy (TKE) distribution of the hydrogen storage tank exhibits a strong, axis-symmetric turbulence pattern caused by the rotation, as shown in Figure 5. The TKE values peak at 38.3 m^2^/s^2^ in the double-ring zones of peak TKE, which are seen in the rounded shear-dominated flow structures. The high shear work done in these regions correlates with the areas of the steepest radial gradients. The calm, central region amid turbulent TKE rings suggests that the rotation draws energy predominantly from the near-wall shear layers to mix the center of the volume to diffuse turbulence throughout the rotating shear layers. There is a significant increase, within the range of 18 to 22 m^2^/s^2^, in the volume-averaged TKE compared to the non-rotating case, which confirms that the contribution of rotation to turbulence generation is strong.

The expected behavior of enhanced mixing is observed in the turbulence field. At 30 rad/s, the flow is sufficient for turbulent transport to disrupt thermal stratification and increase the rate of heat removal. Additionally, the peak values of TKE are low enough to prevent dominant viscous dissipation and counterproductive heating. This identifies 30 rad/s as a potentially optimal operating regime where the benefits of convective and turbulent mixing exceed the detrimental effects of shear heating.

Figure 6 shows stream function distributions in the hydrogen storage tank for rotational speeds of 10 rad/s (left contour) and 50 rad/s (right contour). At 10 rad/s, the flow field contains a symmetrical and organized toroidal vortex structure along the axis of the tank. There are stream function values of 631 kg/s, and there is a smooth concentric pattern that extends radially from the center of the vortex to the walls. This means that rotation at this speed causes a large-scale circulation with low distortion in the flow field, and promotes a relatively large radial mixing. Conversely, at 50 rad/s (right contour), the stream function field is intense and, in contrast with the previous example, stream function values are 1040 kg/s, meaning there is upper circulation that is higher than average. The center of this region is relatively calm (under 200–300 kg/s); there are stream function values of 200–300 kg/s. The upper region has a high concentration of streamlines, which means that rotation is accelerated, and this means that the upper region is where high rotational flow is located. The volume-averaged stream function shows a notable increase, going from about 250–300 kg/s at 10 rad/s to 450–550 kg/s at 50 rad/s. This change really highlights how much rotational speed impacts the overall circulation strength. At 10 rad/s, the flow is stable and provides uniform convective mixing, which is great for efficient heat removal without risking excessive shear. But when you ramp it up to 50 rad/s, the flow shifts to being dominated by localized high-circulation zones. While this boosts mass transport significantly, it can also lead to increased viscous dissipation and unwanted localized heating. These findings suggest that using intermediate rotational speeds strikes a better balance between improved mixing performance and managing shear-induced thermal effects.

Figure 7 illustrates the static temperature distributions in the hydrogen storage tank after the 1000 s refueling process at rotational speeds of 10 rad/s, 30 rad/s, and 50 rad/s (from left to right). At 10 rad/s (the left contour), you can see a clear central hot region with a peak temperature of about 271 K, surrounded by a relatively smooth gradient toward the cooler walls. The temperature field is fairly symmetric, but the core area is noticeably warmer than the zones near the walls, indicating that this lower rotation speed only achieves moderate convective mixing and does not fully eliminate thermal stratification. Moving to 30 rad/s (the middle contour), the temperature profile shows significant improvement. The peak temperature drops to around 256 K, and the central hot spot is much less pronounced. The transition from the warmer core (orange/red) to the cooler boundary layers (green/blue) is much smoother, suggesting that enhanced circulation is driving stronger radial and axial heat transport. This mid-range speed provides the best uniform temperature distribution among the three cases, resulting in the lowest maximum temperature and even better homogeneity. This suggests the best working range for mixing and thermal efficiency.

In contrast, the temperature distribution is poorer at 50 rad/s (right contour). Here, the peak temperature increases to 253 K or even more in some areas (notice the thick red hotspot at the top). The temperature symmetry is also disrupted. Mixing in the core is impressive, but the upper area hot zone is clearly dominant. This indicates the beginning of counterproductive shear heating (viscous heating). This effect will offset some of the mixing that lower speeds provide. Here, we see the most striking example of a non-monotonic trend as a function of rotational speed.

At 10 rad/s, mixing is initiated but is still insufficient to lower the peak temperature at the center. At 30 rad/s, there is the best thermal uniformity and the lowest peak temperature. This confirms that 30 rad/s provides the best mixing thermal uniformity during refueling. At 50 rad/s, the additional input rotational energy becomes localized conductive heating, which again raises the peak temperature and reduces the overall effectiveness.

This behavior underscores the existence of an optimal rotational speed window—around 30 rad/s in the present study—where convective heat redistribution is maximized while viscous dissipation remains under control.

Figure 8 shows the pressure distributions inside the hydrogen storage tank at the end of the 1000 s refueling process for rotational speeds of 10 rad/s, 30 rad/s, and 50 rad/s (from left to right).

At 10 rad/s (left contour), the pressure field remains relatively uniform across most of the domain, with values largely concentrated in the 0.08–0.09 MPa range. The central region exhibits slightly higher pressure (peak ≈ 0.09255 MPa), forming a mild radial gradient toward the walls. This distribution suggests that rotation at this low speed has only a limited influence on pressure homogenization, and the overall pressure buildup is still modest, consistent with the early-to-mid stage of refueling for a large-volume tank.

At 30 rad/s (middle contour), the pressure profile shows a clear increase in both magnitude and spatial variation. Peak values rise to approximately 2.88 MPa, with the majority of the domain falling in the 1.0–2.5 MPa range. When compared to the 10 rad/s case, the contour shows improved and more symmetric pressure distribution, with the high-pressure central zone still well distributed, being more pronounced. This suggests that the total pressure distribution is improved with the 10 rad/s case due to the rotational speeds optimally cooling the tank. This, most likely, is due to the enhanced mixing and convective transport that helps distribute the incoming gas momentum more effectively. At 50 rad/s (right contour), the pressure field becomes noticeably more intense in localized regions. The maximum pressure reaches 1.144 MPa in a concentrated hot spot near the top, while the bulk of the domain lies in the 0.3–1.0 MPa range. The symmetry is somewhat disrupted, and the upper region shows a stronger pressure accumulation compared to the lower parts. This suggests that higher rotation begins to generate localized pressure gradients, possibly linked to intensified flow recirculation and momentum focusing in certain zones.

The average behavior of the pressure fields indicates a non-monotonic development with the increase in rotational speed. At 10 rad/s, the rotation pressure is low and almost uniform. This indicates a lack of convective effect. At 30 rad/s, the rotation pressure field is the most optimal, with the most even distribution and even average pressure. Meanwhile, at 50 rad/s, the pressure field shows localized pressure peaks and rotates too much, and the momentum distribution becomes uneven, resulting in possible flow instabilities. Overall, the data shows that an average rotational speed of around 30 rad/s is the most optimal for pressure equalization during the refueling process, facilitating gas filling while minimizing adverse pressure gradients.

The velocity magnitude contours at the end of the 1000 s refueling process for 10 rad/s, 30 rad/s, and 50 rad/s (from left to right) are seen in Figure 9. The flow is relatively gentle and well-organized at 10 rad/s for the left contour. The velocity magnitudes are found to be at moderate levels, with a maximum of about 54 m/s, concentrated in a central toroidal structure. The velocity magnitudes remain at moderate values, with a maximum velocity of about 54 m/s, and are located mainly in a somewhat toroidal volume. However, the low rotational speed still seems suitable for the MRF method to produce a coordinated swirling motion, which supports the radial transport. However, the overall kinetic energy input is limited, and low velocities are maintained near the walls. When the speed is ramped up to 30 rad/s (middle contour), velocity magnitudes grow by a considerable margin. The peak value stands at about 186 m/s, and the velocity zone occupies a larger annulus while the center maintains only moderate velocities. The pattern also radially intensifies and extends, suggesting a radical rotational shear and the best transfer of momentum from the rotation frame to the fluid. Near-wall velocities also show a marked increase (in some sections, they reach 50–80 m/s), thus leveraging convective mixing and establishing a relatively uniform heat distribution across the domain. However, at a rotation speed of 50 rad/s (right contour), there is a drastic change in the flow field. The velocities were close to 297 m/s, which is almost twice the magnitude recorded at 30 rad/s. These peak velocities form a narrow and fierce ring close to the walls. Shorter velocities are observed in the center (below 100 m/s), but there are very high shearing rates around the walls, with some localized layers with velocities and shearing rates above 200 m/s. This increase in near-wall velocity indicates that viscous dissipation (or shear-induced heating) becomes more apparent at higher rotation rates, hence negating some of the mixing benefits associated with lower rotational speeds. Here, a distinction is evident, where velocity is linked to rotational speed, whereas flow changes markedly as the vortex, which indicates that the core and wall being equal at 10 rad/s generates a situation where the maximum shear is centered on the wall at 50 rad/s. The velocity distribution at the “intermediate” speed of 30 rad/s is perhaps most conducive to enhancing convective heat transfer: its magnitude is “high enough to increase mixing” without fostering too much localized dissipation. The speed regime of 20–40 rad/s appears to be an optimal region for mixing to occur during refueling, optimizing the momentum transfer aspect without subjugating the scenario to unwanted thermal consequences.

Figure 10 presents the rothalpy distribution following the 1000 s hydrogen refueling process for 10 rad/s, 30 rad/s, and 50 rad/s rotational speeds from left to right. Rothalpy has a fairly symmetrical dome shape with lower values on the walls and higher values in the center (approximately 3.33 × 10^5^ J/kg) on the left contour at 10 rad/s. The rothalpy gradient is gently dominated radially, which indicates somewhat organized flow with minimal transport in the circumferential direction at the lower value of rotation speed. The rothalpy variation over the entire area is not sufficiently enhanced to the extent to which the applied rotation speed leads to a major redistribution of energy. At 30 rad/s (middle contour), the rothalpy field is “more uniform radially” (ibid). Peak values reduce to approximately ~4.62 × 10^5^ J/kg, and the rothalpy core with high readings is wider and less distinguished. The transition between the yellow/red core and the green/blue near-wall areas happens over a wider range of values, which suggests better mixing and more effective transport of energy in the circumferential direction. The 30 rad/s scenario offers the best overall solution to the three rothalpy distribution possibilities since any detrimental effects of mild central excess are more than compensated for by the more controlled cooling at the walls. However, at 50 rad/s (as seen in the right contour), rothalpy starts showing signs of degradation. Its peak value climbs again to around 4.59 × 10^5^ J/kg and localizes strongly in the upper area, where it creates a bright red spot (see the right contour). The distribution begins to lose its symmetry, and the upper part of the tank becomes a rothalpy reservoir, while the bottom half remains comparatively cooler. It seems that at higher rotation rates, local viscous dissipation starts to take over in certain areas, resulting in localized energy buildup to offset the better mixing at lower speeds.

The rothalpy patterns show a non-monotonic effect in relation to the increase in rotational speed. At 10 rad/s, there are positive aspects to mixing, but rotation is still inadequate to remove the excess rothalpy in the center. At 30 rad/s, the optimal distribution is achieved, so the peak is minimized, and better uniformity is achieved. Further increase to 50 rad/s results in the return of localized rothalpy peak. This is likely due to the increased shear and viscous heating in the flow region. However, for rothalpy distribution control, 30 rad/s is the best intermediate rotational speed as it efficiently distributes energy and reduces undesirable local energy accumulation.

Practical feasibility, mechanical requirements, and safety implications of tank rotation. This is an excellent and very important point. It should be emphasized that the present MRF simulations are a numerical proxy for enhanced convective mixing and do not imply that the entire tank structure must physically rotate at high angular velocity. In real-world applications, we may need to mention some concepts. These include (i) internal rotating diffusers or propellers used in their place, driven by inflow kinetic energy or small auxiliary motors, and (ii) vortex-forming inlet designs in fixed installations that create large-scale vortex structures without the need to rotate the entire tank while doing so, or (iii) in fixed installations where it is more practical to apply low-frequency oscillation or shaking to the tank externally. These three mechanisms are of significant importance. As an example, achieving the full physical rotation of a large composite type IV tank at 30 rad/s will indeed present significant engineering hurdles, such as designing bearings, managing fatigue in the composite layers, ensuring dynamic balance, coping with increased system complexity and weight, and maintaining liner integrity under centrifugal forces. In addition to the difficulties of this, it is also normal to consider compatible mechanisms. For example, these challenges make direct mechanical rotation impractical for most vehicle applications, but may be more feasible for fixed storage units where sufficient space and infrastructure are available to support specialized mixing systems. We also need to carefully consider safety implications, such as the potential for new failure modes, before or after implementations here. The main value of the present parametric study lies in demonstrating, at the fluid physics level, the existence of a useful operational framework for the purpose of volume-wide forced convection and in providing target performance measurements (velocity, TKE, dissipation rate) that will lead to future hardware design of vortex-generating inlets, internal low-speed rotors, or oscillatory mechanisms.

Internal flow dynamics were analyzed to verify data consistency between velocity fields and temperature distributions. As a result of the analysis, significant data were obtained. The rotation creates a vortex effect that increases mixing between the cold gas core and the heated walls. Thanks to this rotational motion, which effectively disrupts the thermal stratification, the strong correlation between velocity streamlines and temperature contours is confirmed. Therefore, a consistent physical explanation for the enhancement of heat transfer has been achieved.

Here, international standards were used to ensure evaluation within safe limits. Therefore, rotational speeds (10–50 rad/s) were evaluated under the SAE J2601 safety framework, which limits gas temperatures to 85 °C. The stationary state was also observed. While the static state (0 rad/s) provides a baseline function, rotation initially increases the Nusselt number, significantly increasing convective cooling. However, a temperature increase above 30 rad/s is observed. Of course, this has a physical meaning. This is quantitatively attributed to the viscous distribution in the energy equation. At higher speeds, heat production from fluid shear stress exceeds the rate of convective heat removal. Ultimately, 30 rad/s represents the optimal balance between thermal safety and mechanical efficiency.

Industrial Standards and Operational Restrictions: When restrictions come into play, limitations must be drawn according to SAE J2601, which is the internationally valid standard. While this study serves as a basic proof of concept for rotational thermal management, the selected operational parameters were evaluated in the context of the SAE J2601 standard, which defines the safety and performance limits for hydrogen fueling. In order to ensure structural integrity, not exceeding 85 °C constitutes the gas temperature limit value in the type IV tank according to SAE J2601. The inclusion of rotation speeds can produce significant results. The rotation speed range of 10–50 rad/s examined in this study represents a critical operational window. It has been determined that speeds below this range provide insufficient convective mixing to significantly reduce peak temperatures, while speeds exceeding 50 rad/s may cause excessive mechanical stress on the tank’s mounting assembly and increase the energy consumption of the system without a commensurate gain in cooling efficiency. Thus, the proposed range of 10–50 rad/s enabled the production of remarkable results by providing an optimized balance between thermal safety compliance and mechanical feasibility in real-world fueling scenarios.

## 5. Conclusions

This study has demonstrated, through transient real-gas CFD simulations, the potential of rotational mixing as an innovative active thermal management strategy for large-scale type IV polymer composite hydrogen storage tanks during rapid refueling. By employing the Multiple Reference Frame (MRF) approach to simulate tank rotation at speeds of 10, 30, and 50 rad/s in a 20.56 m^3^ volume tank under a 1000 s fast-refueling scenario, we systematically investigated the interplay between enhanced convective heat transfer and viscous dissipation heating.

Key quantitative findings indicate a strongly non-monotonic relationship between thermal performance and rotational speed. At 10 rad/s, we see a relatively uniform temperature profile with minimal extra energy input, which already shows a noticeable improvement over the stationary baseline. The most effective suppression of peak temperatures is achieved at 30 rad/s, where the intensified internal mixing significantly reduces thermal gradients and lowers the maximum temperature by approximately 10–15 K relative to the non-rotating case. However, further increasing the speed to 50 rad/s introduces substantial viscous dissipation, which generates a secondary temperature rise that partially offsets the benefits of improved convection. This non-monotonic behavior is physically rooted in the competition between turbulence-driven heat removal and shear-induced heating: moderate rotation enhances turbulent kinetic energy and convective transport to the colder walls, while excessive rotation amplifies viscous dissipation in high-shear regions near the wall, leading to localized overheating.

Compared to the stationary reference case, the sweet spot for the optimal rotational speed is around 30 (rad)/s. The latter is optimized since the energy spent influences the speed of rotation. Finally, even at peak simulated temperatures, the tank insides do not exceed the SAE J2601 safety threshold of 85 °C (358 K). This gives us a safe shot in terms of the safety of composite materials and their long-term durability. The realizable k-ε turbulence model coupled with the Redlich–Kwong (RK) equation of state and the Multiple Reference Frame (MRF) approach has been thoroughly validated and adopted in a range of high-pressure hydrogen refueling-related numerical investigations [24,25,26,27] and rotating and stirred fluid scenarios. More specifically, the realizable k-ε model has correlated well with experimental temperature profiles during the refueling of a tank with hydrogen. Typically, temperature averages deviate by 2–5 K (or less than 2 °C under best grid resolution and wall treatment) [24,25,28]. This is a preferred choice due to its compromise between accuracy and efficiency of computational effort for such a flow [26]. The RK equation of state models high-pressure hydrogen behavior very well (up to 70 MPa) with very minor deviations (within a 1–2% margin) from the NIST REFPROP data for density and thermodynamic properties within the relevant range [21,27]. The predicted nonlinear dependence of thermal performance upon rotational speed—exhibiting “upside gains” followed by a viscous dissipation penalty at increased rotational speeds—is in line with rotating heat exchangers, stirred chemical reactors, and other related physics. Thus, any improvements in thermal performance achieved at lower rotational speeds are eventually lost to the viscous dissipation penalty at higher speeds. As for CFD, studies regarding hydrogen tanks applying either passive or active approaches, i.e., optimized inlet diffusers, pre-cooling modifications, or better mixing, show peaks in temperature mitigations between 5 and 25 K [24,25,26]. The relative improvements that are observed here (against a stationary case) at a maximum value are close to 10 to 15 K at 30 rad/s. The MRF method, which requires a rotating fluid domain with stationary walls, is a common choice for stirred-tank CFD simulations [18,19,20].

Despite these benefits, practical implementation requires addressing mechanical challenges such as high-pressure rotating seals and system energy overhead. Future research should focus on scaling this approach for various tank geometries and evaluating its potential to reduce reliance on costly external pre-cooling units.

To summarize, rotational refueling is arguably a more effective approach when compared with the traditional stationary-frame filling approach, which suffers from substantial inlet-jet impingement and localized heating. Its success, however, is limited within a certain speed range. For the large-scale tank setup and refueling conditions we examined, 30 rad/s proves to be the sweet spot, achieving the best balance of temperature uniformity and minimal peak temperature while managing viscous heating effectively. This study underscores the promise of rotational mixing as a hardware-free, active thermal management solution for high-pressure hydrogen storage.

## Data Availability

The original contributions presented in the study are included in the article; further inquiries can be directed to the corresponding author.

## Abbreviations/Nomenclature

**Latin Symbols**	
a	Temperature-dependent attraction parameter in the Redlich–Kwong equation of state
b	Co-volume (excluded volume) parameter in the Redlich–Kwong equation of state
E	Total energy (sensible + kinetic) [J/kg]
g	Gravitational acceleration [m/s^2^]
k	Thermal conductivity [W/m·K] or Turbulence kinetic energy [m^2^/s^2^]
p	Pressure [Pa or MPa]
R	Ideal gas constant [J/kg·K]
S_energy	Source term for the energy equation
S_MRF	Momentum source term derived from the moving reference frame (Coriolis and centrifugal forces)
T	Temperature [K]
t	Time [s]
u	Velocity vector [m/s]
V_m	Molar volume [m^3^/mol]
Greek Symbols	
ε	Turbulence dissipation rate [m^2^/s^3^]
ρ	Density [kg/m^3^]
τ	Viscous stress tensor
ΔT	Temperature difference or gradient [K]
APRR	Average pressure ramp rate
CFD	Computational fluid dynamics
HDPE	High-density polyethylene
MRF	Multiple Reference Frame
NIST	National Institute of Standards and Technology
PEMFC	Proton exchange membrane fuel cell
RK-EOS	Redlich–Kwong equation of state
SAE	Society of Automotive Engineers
SOC	State of charge
TKE	Turbulence kinetic energy
